# Experimental Study on the Influence of Rubber Content on Chloride Salt Corrosion Resistance Performance of Concrete

**DOI:** 10.3390/ma14164706

**Published:** 2021-08-20

**Authors:** Danyang Su, Jianyong Pang, Xiaowen Huang

**Affiliations:** 1 State Key Laboratory of Mining Response and Disaster Prevention and Control in Deep Coal Mine, Anhui University of Science and Technology, Huainan 232001, China; sudy_1314@163.com; 2 School of Civil Engineering and Architecture, Anhui University of Science and Technology, Huainan 232001, China; HuangXW0119@163.com

**Keywords:** chlorine salt corrosion, rubber, concrete, mechanical properties, SEM, EDS

## Abstract

In order to enhance the corrosion resistance of concrete to chloride salt, 5% NaCl solution was used to corrode ordinary concrete (OC) and rubber concrete (RC) with 5%, 10%, and 15% rubber content, respectively. By testing the compressive strength, mass, chloride ion concentration at different depths and relative dynamic elastic modulus, the erosion mechanism was analyzed by means of SEM scanning and EDS patterns, and the mechanical properties and deterioration degree of ordinary concrete (OC) and rubber concrete (RC) under the corrosion environment of chloride salt were studied. The results show that: the quality of rubber mixed into concrete increases first and then decreases, and rubber can increase the compressive strength of concrete, improve its internal structure. At the same time, the mechanical properties of concrete in the corrosion environment of chloride salt are improved to a certain extent, and the deterioration degree is reduced. Considering the comprehensive performance of OC and RC in the dry–wet alternation mechanism under chloride salt corrosion, the best content of rubber is 10%.

## 1. Introduction

In the production and use of concrete, sustainability is a crucial issue, mainly including durability, mechanical properties, ecology and economics, etc. [1]. Jorge de Brito et al. [2] investigated and summarized the sustainability issues of concrete, where the aim is to collect and organize the main sustainable strategies and offset the negative impact of concrete in the process of production and use as much as possible, including the durability of concrete.

The durability of concrete in the marine environment is an important hidden danger to the safety of concrete structures. Among them, chloride ion penetration is one of the main reasons for the durability of concrete, and the effect of dry–wet alternation greatly accelerates the penetration of corrosive ions, and the tidal range area becomes the most serious area for concrete structure corrosion [3,4,5,6,7]. Therefore, it is of great significance to carry out research on the effect of concrete’s anti-chloride ion corrosion performance under dry–wet alternation action. Many scholars have carried out various research studies on the corrosion and deterioration performance of concrete under the action of simulating the dry–wet alternation of the chloride salt. Zhang et al. [8] studied the effect of early carbonation curing on the chloride ion penetration resistance of concrete, and the results show that carbonation curing can effectively reduce the corrosion degree of concrete in the marine environment and delay the occurrence of corrosion to a certain extent. Li Yongqiang et al. [9] studied the corrosion resistance of cement-based composites to chloride ion and its micro-structure change, Friedel salt found in corrosion products, and mixing mineral powder in concrete is beneficial to improve the binding capacity of concrete to chloride ions. Thomas et al. [10] studied the influence of mineral admixtures on the chloride ion binding capacity of cement paste, and the results showed that the chloride ion binding capacity of cement paste increased the most after metakaolin was added. Xu Shilang et al. [11] conducted a rapid chloride ion test on ultra-high toughness concrete and analyzed the free chloride ion content. The results showed that ultra-high toughness concrete has better resistance to chloride ion penetration than ordinary concrete.

In recent years, with the rapid development of the economy and society, the global annual sales of automobiles have increased rapidly, resulting in a sharp increase in the number of waste rubber tires each year. Because waste tires are solid wastes that are not easily degraded, the current treatment methods for waste tires are still mainly combustion, landfill and other methods, which cause serious pollution to the environment [12,13]. I.M. Mousaa et al.’s [14] studies have shown that rubber has good thermal stability, flexibility, abrasion resistance, adhesion, chemical resistance and other properties. Many scholars in the field of civil engineering combine the advantages of rubber materials and consider from the perspective of practical engineering problems, mixing rubber into concrete to prepare rubber concrete with special properties. Compared with ordinary concrete, rubber concrete has good properties of fatigue resistance, damping energy consumption, elastic damping, anti-permeability, impact resistance, etc., with broad application prospects [15,16,17,18,19]. On the other hand, promoting the engineering application of rubber concrete can better solve the “black pollution” caused by waste tire rubber, making rubber concrete a hot spot for scholars at home and abroad [20,21]. For this reason, scholars at home and abroad have performed a lot of scientific experimental research on rubber concrete, and have produced fruitful research results, which provide a basis for the feasibility of rubber concrete materials in practical engineering and promote the development of civil engineering disciplines, such as Chen Aijiu et al. [22] who used rubber particles to replace fine aggregate to prepare rubber concrete and conducted impact resistance tests, and found that rubber can effectively improve the impact resistance of concrete and improve the brittleness of concrete. Meanwhile, Ren Rui et al. [23] studied the influence of rubber particle size and content on the axial compressive performance of concrete, and gave the relationship between the axial compressive strength and cubic compressive strength of rubber concrete, as well as the stress–strain curve and peak stress formula. Kristina Strukar et al. [24] discussed the influence of rubber content on the mechanical behavior of concrete through a uniaxial compression test of rubber concrete, and conducted a comprehensive study on its stress–strain curve, and found that rubber can enhance the ductility of concrete. Katherine E et al. [25] used acoustic emission technology to study the wear resistance of ordinary concrete and rubber concrete, and found that the weight loss and wear depth of rubber concrete are significantly lower than that of ordinary concrete. Ruonan Zhu et al. [26] studied the chlorine salt corrosion resistance of rubber concrete in 4% NaCl solution, which has certain guiding significance for the chlorine salt corrosion resistance of rubber concrete. Jian Liang et al. [27] showed that under the action of chloride ion corrosion, rubber can delay the development of concrete cracks and reduce the peak value of steel corrosion. Han Zhu et al. [28] studied the chloride ion penetration resistance of rubber concrete under different environmental temperatures, and found that rubber can reduce the corrosion degree of reinforcement in concrete, and the durability of rubber concrete changed with different environmental temperatures. Han Qinghua et al. [29] systematically tested and calculated the chloride ion transport and erosion mechanism of rubber concrete from both macroscopic and microscopic simulations. The results show that rubber can effectively reduce the chloride ion diffusion coefficient and improve the durability of concrete structures.

Based on the serious durability deterioration of concrete caused by chloride ion penetration in ocean engineering and the current application of rubber in the field of civil engineering, this paper designed and applied 5% NaCl solution under the action of dry–wet alternation to simulate the ocean tidal environment. The compressive strength, mass, chloride ion concentration at different depths and relative dynamic elastic modulus of rubber concrete with different contents were tested, and SEM and EDS tests were carried out to analyze the difference of chloride ion permeability resistance between ordinary concrete and rubber concrete under dry–wet alternation action, in order to provide a reference for relevant engineering practices.

## 2. Materials and Methods

### 2.1. Raw Materials

The cement used in the test is P·O42.5 grade ordinary Portland cement, and the performance indicators are shown in Table 1. Continuous graded crushed stone with particle size not less than 5 mm is used as coarse aggregate. The fine aggregate is natural river sand, which belongs to medium sand, and the fineness modulus is 2.55. Rubber particle size is 20 mesh and density is 1050 kg/m^3^. For NaCl, analytical reagent type anhydrous sodium chloride was selected. A high-performance water reducer (HPWR) with a water-reducing rate of 37% is employed to guarantee fluidity and water retention.

### 2.2. Mix Proportion Design

We refer to the relevant regulations in JGJ 55-2011 *Specification for mix proportion design of ordinary concrete* to design the mix proportion of ordinary concrete (OC) and rubber concrete (RC) in this paper. In this test, four gradients of rubber content are 0%, 5%, 10%, and 15% of the mass of fine aggregate are used, and the water reducer dosage is determined according to the working ability. The specific mix proportions of OC and RC are shown in Table 2.

### 2.3. Specimen Preparation

We use a single-shaft horizontal concrete mixer to make specimens. First, pour the weighed stones and sand into the mixer and dry mix for 1 min, then pour in rubber and cement and continue dry mix for 1 min, and finally add weighed water dissolved with water reducer and stir for 3 min. Then the mixed concrete is put into the concrete mold and vibrated on the vibrating platform. After 24 h, the mold was removed, and OC and RC were made. After the completion of the specimen, indoor standard curing was carried out under natural conditions for 28d. The curing temperature range was 16–24 °C, and the humidity was more than 95%. The compressive strength was tested with the size of 100 mm × 100 mm × 100 mm, and dynamic compression performance tests used cylindrical specimens with a diameter of 74 mm and a height of 38 mm.

### 2.4. Test Plan

The chloride salt corrosion test program is carried out in accordance with the relevant regulations in the National Standard of the People’s Republic of China GB/T 50082-2009 Standard for test methods of long-term performance and durability of ordinary concrete. The concentration of the NaCl solution is 5%. Put the specimens cured for 28 days into the prepared NaCl solution. The distance between adjacent specimens during erosion should not be less than 20 mm, and the solution should exceed the upper surface of the uppermost specimen by at least 20 mm, so that the specimens can be fully eroded. The drying temperature is 85 °C, the dry–wet alternate time is 72 h, and the dry–wet ratio is 3:1. The dynamic modulus of elasticity, compressive strength, chloride ion concentration at different depths and mass of the specimens of each group were tested, respectively, at 0, 15, 30, 45, 60, 75 and 90 times of dry–wet alternation.

In accordance with the relevant regulations in the National Standard of the People’s Republic of China GB/T 50081-2019 Standard for test methods for concrete physical and mechanical properties, the WAW-2000D electro-hydraulic servo universal testing machine is used to carry out the cubic compressive strength test and the calculation method of compressive strength value is shown in formula (1).

(1) $$f_{cc}=\frac{F}{A}$$

where *f_cc_* is the cubic compressive strength of the specimen (MPa); *F* is the failure load of the specimen (N); *A* is the pressure-bearing area of the specimen (mm^2^).

Measure the mass of OC and RC specimens and calculate the mass loss rate in accordance with the relevant regulations in the National Standard of the People’s Republic of China GB/T 50082-2009 Standard for test methods of long-term performance and durability of ordinary concrete. The calculation method is shown in formula (2).

(2) $$W=\frac{W_{0i}-W_{ni}}{W_{0i}}\times 100$$

where *W* is the mass loss rate (%) of the *i*-th concrete specimen after the dry–wet alternation test; *W_0i_* is the mass of the *i*-th concrete specimen before the dry–wet alternation test (kg); *W_ni_* is the mass of the *i*-th concrete specimen after *n* times of dry–wet alternation test (kg).

The HDM-1A concrete grinder was used to stratified sampling the chloride salt corroded concrete specimens under different dry–wet alternations. The powders with different grinding depths were collected and passed through a 0.16 mm square hole sieve. The collection depths were 0–6 mm, 6–12 mm, 12–18 mm, 18–24 mm and 24–30 mm, respectively. The free chloride ion concentration of concrete adopts the water-soluble extraction method [30]; weigh 4 g of sieved powder and dissolve it in 40 g of extraction solution. The total chloride ion concentration adopts the acid-soluble extraction method; weigh 5 g of the sieved powder and dissolve it in 40 g extraction solution, fully shake it for 5 min and then stand for 24 h. The DY2501-B rapid measuring instrument for chloride ion content is used to determine the free chloride ion and total chloride ion concentration.

The test of the dynamic elastic modulus of concrete is carried out in accordance with relevant regulations in the National Standard of the People’s Republic of China GB/T 50082-2009 Standard for test methods of long-term performance and durability of ordinary concrete, using the DT-W18 dynamic modulus of elasticity tester for testing.

First, measure the mass and size of the specimen to ensure that the connections and relative positions of the components of the dynamic modulus of elasticity tester meet the requirements of the specification. Then, adjust the tester to make the specimen reach resonance state, and use the resonance frequency displayed at this time as the specimen fundamental frequency vibration frequency. The calculation formula of dynamic elastic modulus is shown in formula (3).

(3) $$E_{d}=13.244\times 10^{-4}\times \frac{WL^{3}f^{2}}{a^{4}}.$$

where *E_d_* is the dynamic elastic modulus of the specimen (MPa); *a* is the side length of the section of the cube specimen (mm); *L* is the length of the specimen (mm); *W* is the mass of the specimen (kg), accurate to 0.01 kg; *f* is the fundamental vibration frequency of the specimen when it transverse vibration (Hz).

The relative dynamic elastic modulus calculation formula is shown in formula (4).

(4) $$E=\frac{E_{di}}{E_{0i}}.$$

where *E* is the relative dynamic elastic modulus of the specimen; *E_di_* is the dynamic elastic modulus of the *i*-th concrete specimen after the dry–wet alternation test (MPa); *E_0i_* is the dynamic elastic modulus of the *i*-th concrete specimen before the dry–wet alternation test (MPa).

## 3. Test Results and Analysis

### 3.1. Compressive Strength

**Figure 1:**
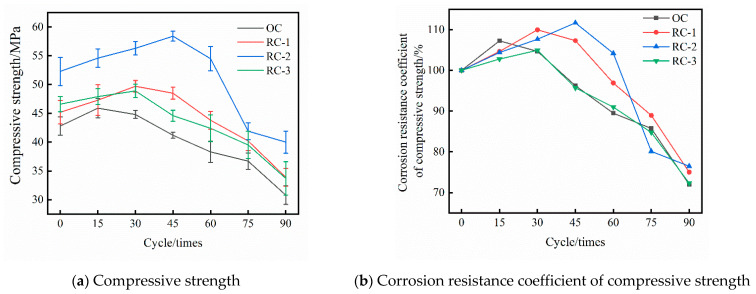
The compressive strength of OC and RC and the corrosion resistance coefficient of compressive strength under dry–wet alternation in the chloride salt erosion environment. (**a**) Compressive strength, (**b**) Corrosion resistance coefficient of compressive strength.

Figure 1a shows the compressive strength of OC and RC in different periods of dry–wet alternation in a chloride salt erosion environment. The following can be seen intuitively from Figure 1a: (1) The compressive strength of the four groups of specimens cured for 28 days under standard conditions of 0 times of dry–wet alternation shows a trend of first increasing and then decreasing with the increase in rubber content, which shows that rubber can improve the compressive strength of standard cured 28d concrete to a certain extent; (2) RC performs better than OC in compressive strength under the action of dry–wet alternation in the corrosion environment of chloride salt; (3) In the corrosion environment of chloride salt, with the dry–wet alternation conditions, the compressive strengths of OC and RC both show a “two-stage” mode that first increases rapidly and then decreases. When the dry–wet alternate is at 15 times, the OC compressive strength begins to show a downward trend. The compressive strength of the concretes of RC-1 and RC-3 groups began to decline after 30 times of dry–wet alternation, while the compressive strength of RC-2 group concretes began to decline after 45 times of dry–wet alternation. The peak compressive strengths of the OC, RC-1, RC-2, and RC-3 groups in the corrosion environment of chloride salt with different periods of dry–wet alternation were 45.9 MPa, 49.7 MPa, 58.4 MPa, and 48.9 MPa. After the peak compressive strength of the above groups of specimens appeared, the compressive strength decreased rapidly as the dry–wet alternation continued. When the dry–wet alternation was at 90 times, the compressive strength of the OC, RC-1, RC-2, and RC-3 groups was 30.8 MPa, 33.9 MPa, 40.0 MPa, and 33.7 MPa.

Figure 1b is the corrosion resistance coefficient line chart of compressive strength of the four groups of concrete. At the initial stage of dry–wet alternation between OC and RC, chlorine salt erosion products fill the internal pores of concrete. According to the principle of damage mechanics, with the effect of dry–wet alternation and the depth of concrete hydration, the compactness of concrete is enhanced, and the effective bearing area is also increased, thus the compressive strength is enhanced. The OC, RC-1, RC-2, and RC-3 groups reached the “inflection point” at 15 times, 30 times, 45 times, and 30 times of dry–wet alternations, respectively. At this time, the corrosion resistance coefficient of compressive strength of OC, RC-1, RC-2, and RC-3 groups is increased to 107.24%, 109.96%, 111.66%, 104.94%. After the “inflection point” of each group of concrete, under the combined action of crystallization pressure and swelling pressure, the erosion thickness of the outer surface of the concrete is increased, the pores expand rapidly to form microcracks, the internal erosion layer of concrete gradually cracks and peels off, and the compressive strength decreases rapidly. When the dry–wet alternation was at 90 times, the corrosion resistance coefficient of compressive strength of OC, RC-1, RC-2, and RC-3 groups were 71.96%, 75.0%, 76.48%, 72.32%.

In summary, it can be seen that: on the one hand, under the action of dry–wet alternation in the corrosion environment of chloride salt, RC compressive strength and corrosion resistance coefficient of compressive strength are better than OC, which shows that the incorporation of rubber into concrete can improve the compressive strength of concrete in the environment of chloride salt erosion. On the other hand, the RC-2 group can better delay the decrease in the concrete compressive strength than the R-1 group and the R-3 group, and at the same time, the corrosion resistance coefficient of compressive strength is higher. If only from the perspective of improving the compressive strength and the corrosion resistance coefficient of compressive strength of concrete in the chloride salt erosion environment under the action of dry–wet alternation, it is recommended that the rubber content should be 10%.

### 3.2. Mass

**Figure 2:**
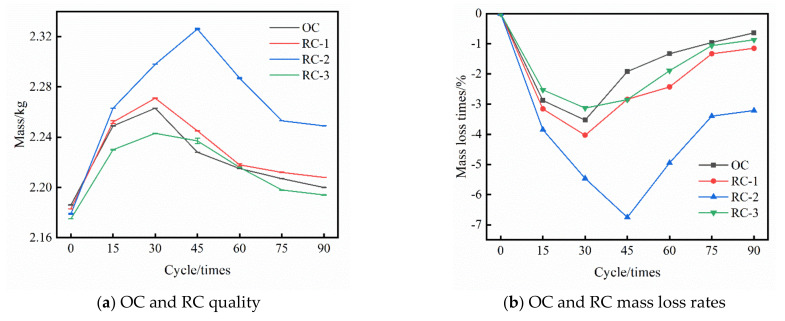
Mass and mass loss rate of OC and RC under dry–wet alternation in the chloride salt erosion environment. (**a**) OC and RC quality, (**b**) OC and RC mass loss rates.

Figure 2a,b, respectively, show the mass and mass loss rate of OC and RC under different dry and wet cycles in the chloride salt erosion environment. It can be seen from Figure 2 that the mass change of OC and RC through 90 times of dry–wet alternation effects in the chloride salt environment can be divided into four main stages: rapid rise stage, slow rise stage, slow decline stage and continuous stable stage. In the initial stage of drying–wetting alternation in chloride environment, chloride ion reacts with 3CaO·Al_2_O_3_ and 4CaO·Al_2_O_3_·Fe_2_O_3_ inside the concrete to form Friedel‘s salt (3CaO AlO_3_·CaCl_2_·10H_2_O) and calcium oxide, which quickly fill the pores on the surface of the concrete. The mass of OC and RC increased rapidly, and the mass-loss rate decreased rapidly. When the dry–wet alternation to 30 times, the mass of OC group, RC-1 group and RC-3 group appeared “inflection point”. At this time, the mass of three groups of concrete was 2.263 kg, 2.271 kg and 2.243 kg, and the mass-loss rates were −3.53%, −4.03% and −3.13%. When the dry–wet alternation to 45 times, the mass of RC-2 group appeared “inflection point”, the mass and mass-loss rates were 2.326 kg and −6.75%. After that, due to the intensification of hydration reaction and the reaction between Cl^−^ and calcium hydroxide in the pores, a large number of expansive compound salt CaCl_2_·Ca(OH)_2_·H_2_O is generated, which will cause the expansion pores in the concrete, resulting in cracks on the surface of concrete and long cracks. At the same time, salt crystallization will also precipitate under the action of high-temperature drying. The generated expansion pressure makes the concrete crack produce microcracks, resulting in the slow decline of the mass of OC and RC, and the slow increase in the mass-loss rate. Finally, it reaches a stable stage after 75 times of dry–wet alternation. When the dry–wet alternation was at 90 times, the mass of OC, RC-1, RC-2 and RC-3 groups was 2.2 kg, 2.208 kg, 2.249 kg and 2.194 kg, and the mass loss rates were −0.64%, −1.15%, −3.21% and −0.87%.

### 3.3. Chloride Ion Content

**Figure 3:**
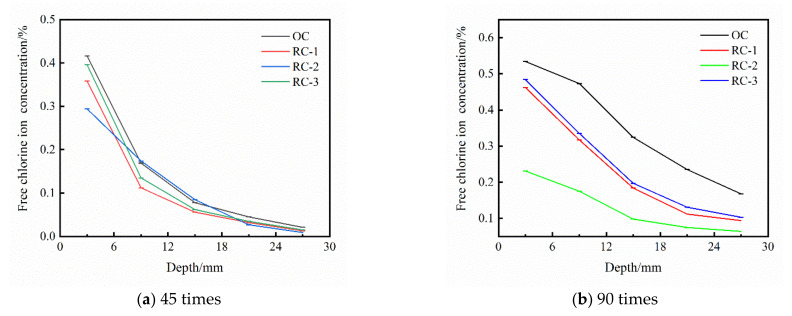
OC and RC free chloride ion concentration at 45 and 90 times of dry–wet alternation in chloride salt erosion environment. (**a**) 45 times, (**b**) 90 times.

The free chloride ion concentrations of OC and RC at different depths are shown in Figure 3 at 45 and 90 times of dry–wet alternation. It can be seen intuitively: (1) The free chloride ion concentration in OC and RC specimens decreases with the increase in depth under the action of dry–wet alternation in chloride salt erosion environment. (2) The free chloride ion concentration in RC specimens decreased compared with OC specimens.

It can be seen from Figure 3 that the incorporation of rubber into concrete significantly reduces the free chloride ion concentration in different depths of concrete, and the anti-chloride ion erosion ability of concrete is in the order of RC-2 group > RC-1 group > RC-3 group > OC group, which indicates that the appropriate amount of rubber incorporation into concrete can improve the resistance to chloride ion erosion of concrete and reduce the intrusion of chloride ion, but the effect of excessive rubber incorporation into concrete is poor. If only from the perspective of reducing the free chloride ion concentration in different depths inside the concrete, it is suggested that the rubber content is 10%.

Table 3 shows the chloride ion binding rate of concrete at different depths when dry–wet alternation 90 times in the chloride salt erosion environment. It can be seen that the chloride ion binding capacity of RC is higher than that of OC when the concrete depth is less than 12 mm, and the RC-2 group has the best chloride ion binding capacity. This shows that rubber can effectively enhance the ability of concrete to bind to chloride ions and improve the ability of concrete to anti-chloride ions corrosion. When the depth exceeds 12 mm, there is no significant difference in the binding capacity of chloride ions between OC and RC due to the low chloride concentration.

### 3.4. Relative Dynamic Elastic Modulus

**Figure 4:**
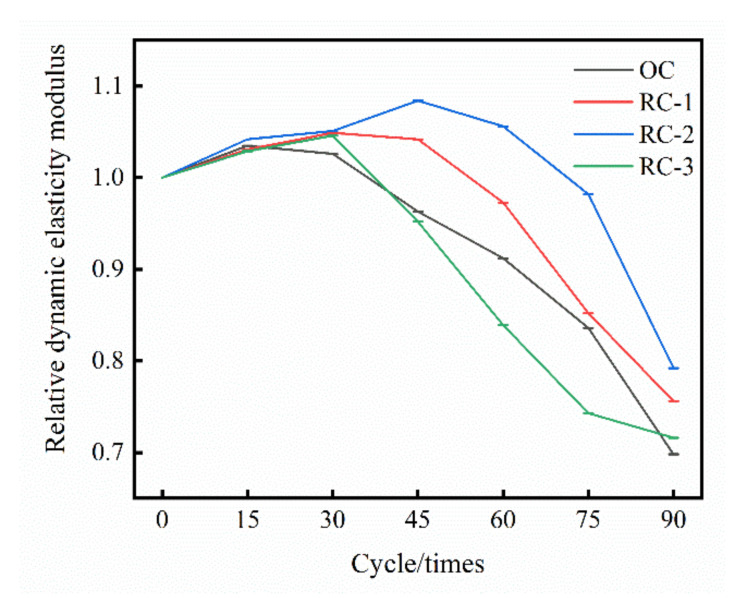
Relative dynamic elastic modulus of OC and RC.

The relative dynamic elastic modulus of OC and RC under the dry–wet alternation action in chloride erosion environment is shown in Figure 4. It can be seen intuitively that the relative dynamic elastic modulus of OC and RC both increase first and then decrease with the dry–wet alternation cycle. The relative dynamic elastic modulus of OC and RC increased rapidly in the initial stage because the products of chloride salt and cement hydration products and the crystals formed by chloride salt filled the pores in the concrete, making the concrete denser; with the dry–wet alternation progress, the expansion and crystallization of the corrosion products cause more cracks in the concrete, resulting in more chloride salt infiltration into the concrete, accelerate the rate of concrete erosion, and the dynamic elastic modulus decreases rapidly.

It can be seen from Figure 4 that, compared with OC, the relative dynamic elastic modulus of RC increases faster in the initial stage, and decreases slowly in the later stage of the dry–wet alternation. This indicates that mix rubber into the concrete is beneficial to reduce the pores in the concrete, enhance the compactness of the concrete, and enhance the ability of concrete’s anti-chloride salt corrosion. When the dry–wet alternation reaches 15 times, the OC dynamic elastic modulus reaches the peak value, increasing by 3.5%; when the dry–wet alternation reaches 30 times, the dynamic elastic modulus of the RC-1 group and the RC-3 group reached the peak value, increasing 4.9% and 4.6%, respectively; when the dry–wet alternation reaches 45 times, the dynamic elastic modulus of the RC-2 group reached the peak value, an increase of 8.4%. It can be seen that the R-2 group of specimens has the smallest degree of degrading and damage under the dry–wet alternation action in a chloride salt corroded environment.

## 4. SEM Micro and EDS

**Figure 5:**
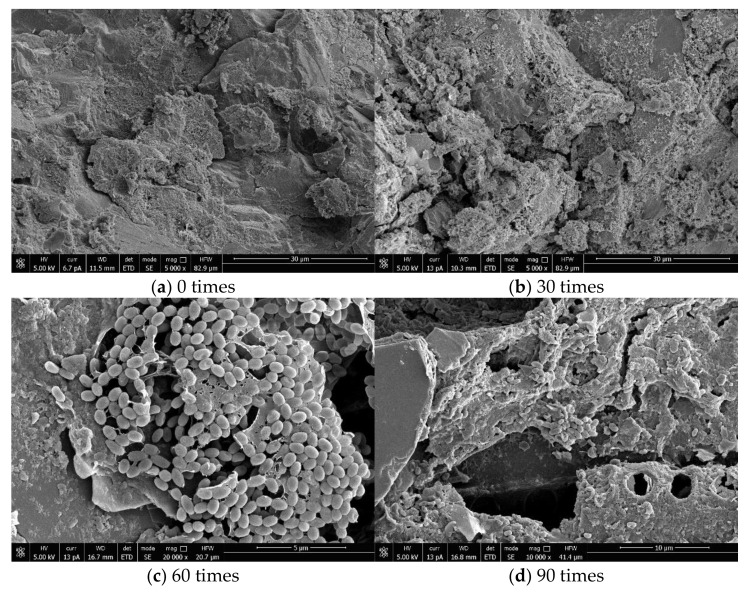
SEM photos of OC group at 0 times (**a**), 30 times (**b**), 60 times (**c**), and 90 times (**d**) of dry–wet alternation in chlorine salt erosion environment.

**Figure 6:**
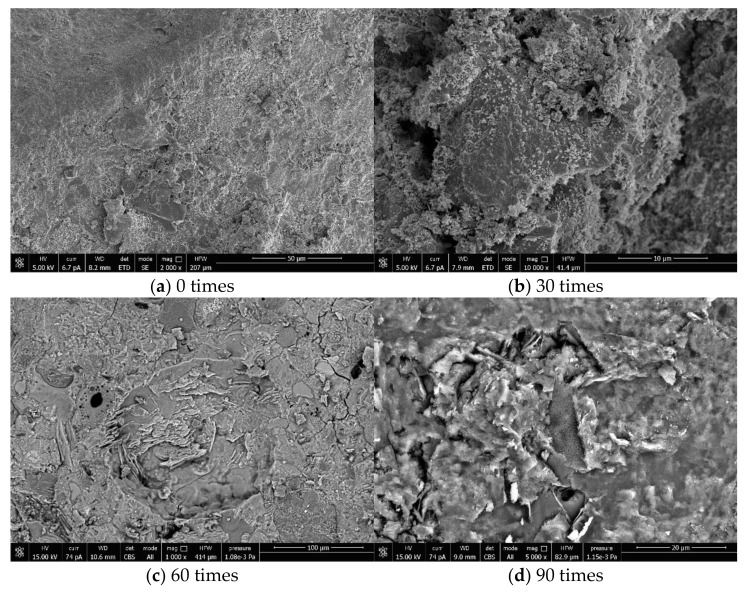
SEM photos of RC-2 group at 0 times (**a**), 30 times (**b**), 60 times (**c**), and 90 times (**d**) of dry–wet alternation in chlorine salt erosion environment.

Figure 5 shows the internal micromorphology of the specimens in the OC group in the chlorine salt erosion environment with 0, 30, 60, and 90 times of dry–wet alternation. Figure 6 shows the internal micromorphology of the specimens in the RC-2 group in the chloride salt erosion environment with 0, 30, 60, and 90 times of dry–wet alternation. Compared with the internal micromorphology of the OC group and the RC group when the dry–wet alternation 0 times, it can be seen that the overall structure of the OC is relatively loose and porous with more pores on the surface. The connection between the aggregate and the adhered mortar is not tight enough, there is an obvious interface transition zone and there are some cracks around the interface, while the surface of RC is relatively smooth, the aggregate particles are regular and tidy, there are fewer pores, and the structure is compact. It can be seen from Figure 5 and Figure 6 that with the dry–wet alternation progress, the pore on OC and RC surface increases, the structure is more loose, and a large number of salt crystals are attached. This shows that after the chloride ion penetrates into the concrete, it chemically combines with the concrete to form Friedel’s salt and expansive compound salt, and these two reactions violently consume the internal hydroxide radicals in the concrete, causing the decomposition of C-S-H, degrading the pore structure of the concrete, and reducing the compactness and stability of the concrete. In particular, OC has a high adhesive mortar content, weak bonding at the interface, and many internal pores and cracks in the interior. A large number of pores accelerate the penetration of chloride ions in the concrete, resulting in the looser internal structure of OC. In contrast, the internal structure of RC is more dense than that of OC.

**Figure 7:**
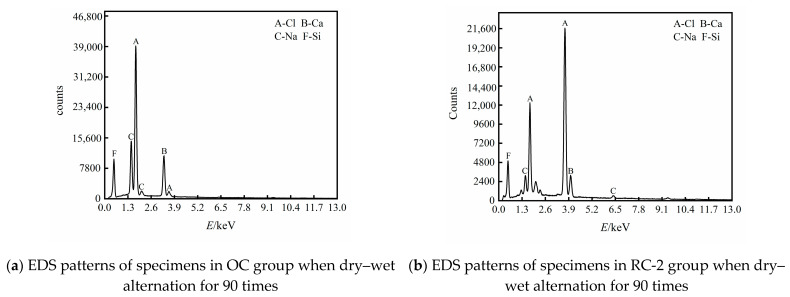
EDS patterns of specimens in OC group and RC-2 group when they are corroded by chloride salt for dry–wet alternation for 90 times. (**a**) EDS patterns of specimens in OC group when dry–wet alternation for 90 times, (**b**) EDS patterns of specimens in RC-2 group when dry–wet alternation for 90 times.

The EDS patterns of the specimens in the OC group and the RC-2 group are shown in Figure 7 when they are corroded by chloride salt for dry–wet alternation for 90 times. It can be seen from Figure 7 that the chloride element in the RC-2 group is significantly lower than that in the OC group. This is because the rubber is mixed into the concrete effectively improve the binding capacity of concrete to chloride ion and reduces the chloride ion concentration in the concrete.

## 5. Conclusions

(1) The compressive strength and relative dynamic elastic modulus of OC and RC both increase first and then decrease with the increase in dry–wet alternation times. The “inflection point” of compressive strength and corrosion resistance coefficient of OC, RC-1, RC-2, and RC-3 groups is 15 times, 30 times, 45 times, and 30 times dry–wet alternation times, respectively. At this time, the corrosion resistance coefficient of compressive strength of OC, RC-1, RC-2, and RC-3 groups is increased to 107.24%, 109.96%, 111.66%, 104.94%, respectively. The addition of rubber into concrete is beneficial to improve the compressive strength of concrete and the compactness of concrete in a chloride salt corroded environment.
(2) The mass changes of the OC and RC groups are mainly divided into the rapid rising stage, slow rising stage, slow declining stage and continuous stable stage. When the dry–wet alternation reaches 30 times and 45 times, the mass of OC group, RC-1 group, RC-3 group and R-2 group appears as an “inflection point”. Under the combined action of the crystallization pressure and the swelling stress, the pores rapidly expand and form micro-cracks.
(3) Adding rubber into concrete can effectively improve the resistance of concrete to chloride erosion and delay the erosion effect of chloride ions on specimens. When the rubber content is 10% of fine aggregate, it has the most obvious improvement effect on the resistance to chloride erosion of concrete. Comprehensive SEM and EDS analyses show that rubber can effectively reduce the porosity of concrete, improve the bonding ability of chloride ions, and reduce the influence of chloride erosion on concrete.

## Figures and Tables

**Table 1:** Cement performance indicators.

Soundness	Specific Surface Area/(m^2^·kg^−3^)	Ignition Loss/%	Alkali Content/%	Setting Time/min		Compressive Strength/MPa	
				Initial Set	Final Set	3d	28d
1.2	316	4.1	0.37	135	270	20.9	42.5

**Table 2:** Mix proportion of OC and RC kg/m^3^.

Specimen	Cement	Coarse Aggregate	Fine Aggregate	Rubber	Water	Water Reducing Agent
OC	430	1185	650	0	195	5.25
RC-1	430	1185	617.5	32.5	195	5.25
RC-2	430	1185	585	65	195	5.25
RC-3	430	1185	552.5	97.5	195	5.25

**Table 3:** Chloride ion binding rate of concrete at different depths when dry–wet alternation 90 times.

Specimen Number	0–6 mm	6–12 mm	12–18 mm	18–24 mm	24–30 mm
OC	0.72	1.35	4.03	4.20	4.31
RC-1	0.82	2.33	4.02	4.29	4.48
RC-2	0.98	2.45	4.03	4.38	4.52
RC-3	0.79	1.96	4.02	4.25	4.37

## Data Availability

The data used to support the findings of this study are available from the corresponding author upon request.
